# The Risk of Very Late-Onset Schizophrenia Following Diabetes Type 2 Onset: A Nationwide Population-Based Study of Midlife and Old-Age

**DOI:** 10.1093/schbul/sbaf159

**Published:** 2025-09-13

**Authors:** Stephen Z Levine, Arad Kodesh, Abraham Reichenberg

**Affiliations:** School of Public Health, University of Haifa, Haifa, 3103301, Israel; Department of Community Mental Health, University of Haifa, Haifa, 3103301, Israel; Mental Health, Meuhedet Health Services, Tel Aviv, 6203854, Israel; Department of Psychiatry, Icahn School of Medicine at Mount Sinai, New York, NY, 10029, United States; Department of Environmental Medicine and Public Health, Icahn School of Medicine at Mount Sinai, New York, NY, 10029, United States

**Keywords:** schizophrenia, diabetes, reverse causation, epidemiology, risk, endocrinology

## Abstract

**Background and Hypothesis:**

Schizophrenia is an established Type 2 Diabetes (T2D) risk factor; while the reverse hypothesis is plausible, it remains untested.

**Study Design:**

This nationwide cohort study included all members (n = 99 567; Female: 52517, 52.7%) of a non-profit Israeli health maintenance organization born between 1932 and 1952. At cohort entry (aged M = 59.70, SD = 5.68) without histories of T2D or schizophrenia, the cohort was followed-up on average 14.47 (SD = 2.28) years for incident schizophrenia. Cox regression models were fit to quantify the association between T2D and schizophrenia risk with the Hazard Ratio (HR) and their 95% Confidence Intervals (CI), unadjusted and adjusted for 20 potential confounders in the primary analysis.

**Study Results:**

During follow-up, schizophrenia incidence per 10 000 person-years was 0.26 (95% CI, 0.21-0.32) in individuals with T2D and 0.12 (95% CI, 0.11-0.14) in those without. In the primary analysis, T2D onset was associated with a 50% increased risk of incident schizophrenia (adjusted hazard ratio = 1.53; 95% CI, 1.11–2.10; *P* = .009) compared with the absence of T2D. Generally, nine complementary analyses were consistent with the primary analysis results, showing T2D was associated with an increased risk of incident schizophrenia; the association showed minimal reverse causation and antidiabetic medication was not associated with schizophrenia risk.

**Conclusions:**

In this study, the onset of T2D was associated with an increased risk of schizophrenia. This suggests that the onset of T2D may require psychosis monitoring, which is relevant to healthcare providers and clinicians in psychiatry, geriatrics, and endocrinology.

## Introduction

Individuals with schizophrenia have a two- to three-fold higher risk for developing type 2 diabetes (T2D) compared to the general population, and after developing T2D, a three- to four-fold higher mortality rate than those with T2D only.^1^^,^^2^ Consensus exists that schizophrenia is associated with an increased risk of T2D.^3^ Among the widely discussed reasons proposed for this association are atypical antipsychotic medication use,^4^ and the increased presence of known risk factors for T2D in individuals with schizophrenia, especially obesity, poor diet, and sedentary lifestyle.^5^

Nevertheless, several lines of evidence suggest that the association between T2D and schizophrenia may also be reversed: T2D may be associated with an increased risk of schizophrenia. First, in schizophrenia patients, the prevalence of diabetes is elevated in the year before the diagnosis of schizophrenia.^6^ Second, in antipsychotic naïve first-episode psychosis patients, there is an increased prevalence of various abnormal glucose markers.^7^^,^^8^ Third, the risk of T2D is increased in siblings of individuals with schizophrenia.^9^ Furthermore, family histories of diabetes and schizophrenia were found to be associated, suggesting that shared familial risk factors may be at play.^10^ A meta-analysis estimated that a family history of T2D was associated with more than four-fold higher odds of comorbid T2D in patients with non-affective psychosis.^11^ Four, one study reported that a well-known T2D genetic risk variant, TCF7L2, was associated with an increased schizophrenia risk.^12^ Five, genome-wide studies suggest a shared genetic risk between schizophrenia and T2D. Polygenic risk score and colocalization analyses suggest that individuals with comorbid schizophrenia and T2D carry a higher genetic predisposition to both disorders, with evidence of overlapping genomic regions and biological pathways.^13^ Collectively, this evidence is consistent with the hypothesis that T2D is associated with an increased schizophrenia risk.

The current study aims to test the hypothesis that incident T2D is associated with an increased risk of incident schizophrenia in middle and old age using a nationwide cohort study design. In complementary analyses, we address potential sex differences, schizophrenia risk modification by anti-diabetic medication,^7^ schizophrenia-onset subtypes, the diagnostic reliability of schizophrenia, clinical subtypes of adult-onset schizophrenia,^14^ and reverse causation^15^ (Figure S1).

## Methods

### Population

The current study source population is based on electronic health records maintained at “Meuhedet Healthcare Services” (henceforth Meuhedet). Meuhedet provides nationwide healthcare services to 14% of the population of the State of Israel.^16^ Meuhedet is one of four non-profit national healthcare maintenance organizations (HMOs) in Israel that provide national healthcare based on legislation.^17^ This legislation makes it illegal for HMOs to deny membership based on demographic factors, health conditions, or medication needs. Hence, this legislation limits selection bias in the current study. The current study cohort was initially established with the overall aim of examining diseases of aging. The Institutional Review Board at the University of Haifa and the Meuhedet Helsinki Institutional Review Board granted ethical approval to conduct the current study with a waiver of written informed consent because the research data were anonymized.

### Study Design

**Figure 1:**
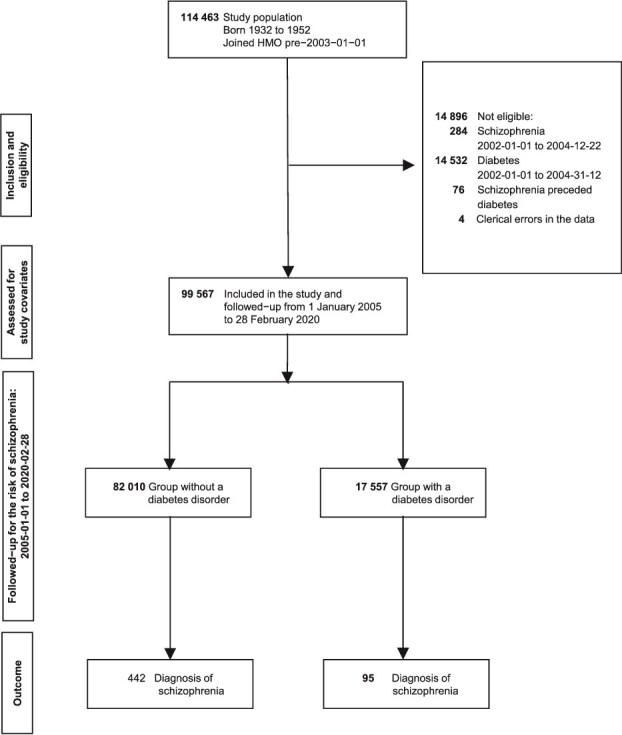
Study Flow Diagram of Cohort Selection, Inclusion, Exclusion, and Follow-Up.

A nationwide historical cohort study was implemented (Figure 1). The source population was all Israeli citizens who were all Meuhedet members nationwide born from 1932 to 1952. Prior to delayed cohort entry on 1 January 2005, participants with a history of a diabetes diagnosis or medication or a schizophrenia spectrum disorder from 2002 to 2004 were not eligible for inclusion in the current study analytic cohort (Figure 1). The analytic cohort was followed up from 1 January 2005 (mean [SD] age, 59.70 [5.68] years) for incident T2D, incident schizophrenia, death, leaving the HMO, or the end of the study follow-up on 28 February 2020 (mean [SD] age, 74.17 [5.65] years). The choice of the end of follow-up date was determined by the COVID-19 pandemic that began in May 2020 in Israel and may have differentially altered the study exposure, outcome, and covariate rates.

### Exposure: Ascertainment of Type 2 Diabetes

The diagnosis of incident T2D was classified as a time-dependent covariate (Figure S2) ascertained from ICD-9 codes from 1 January 2002 to 28 February 2020 (Table S1). Participants with all-cause diabetes diagnoses^18^ (ICD-9: 250^*^) from 01 January 2002 to 31 December 2004 were not included in the study (Figure 1). This data source has been the topic of prior diabetes studies.^19^

### Outcome: Ascertainment of Incident Very Late-Onset Schizophrenia

The diagnoses of Schizophrenia Spectrum Disorder are classified based on the International Classification of Diseases Ninth Revision (ICD-9) (codes: 295^*^)^18^ and the International Statistical Classification of Diseases and Related Health Problems, Tenth Revision^20^ (ICD-10; codes: F20–F29) at Meuhedet. Diagnoses of schizophrenia are made by a medical board-certified psychiatrist. To identify incident cases of adult schizophrenia, individuals were excluded from the study with a schizophrenia diagnosis from 01 January 2002 to 31 December 2004 (Figure 1). These schizophrenia diagnoses have been the topic of prior research.^21^ In complementary analyses, the outcome was redefined to follow-up to the risk of late-onset schizophrenia (onset age 50–60 years) and very-late-onset schizophrenia-like psychosis (onset >60 years).^13^

### Covariates

The study covariates were chosen to account for the possibility that a confounding factor may explain the study association (although more modern DAG-based definitions of a confounder exist).^22^ These were background information, comorbid health conditions, and antidiabetic medications. The background information was age at cohort entry in 2005 (entered as linear and quadratic terms),^23^ sex (female and male), and neighborhood socioeconomic status (SES; classified as low, medium, and high).^24^ Smoking status, often ascertained in medical visits owing to the high smoking rate in Israel,^25^ was a time-varying covariate (Figure S2), classified as present from the report of smoking to the end of follow-up; otherwise, it was classified as absent. Similarly, comorbid health conditions were time-dependent covariates and classified as present from the first diagnosis to the end of follow-up; otherwise, they were absent. These were: Malnutrition (ICD-9: 263^*^), Obesity (ICD-9: 278.0^*^), Asthma (ICD-9: 493^*^), COPD (ICD-9: 496), Hypertension (ICD-9: 401-405), Ischemic Heart Disease (ICD-9: 410-414), Heart failure (ICD-9: 428^*^), Atrial fibrillation (ICD 9: 427.31), Cerebrovascular Disease (ICD 9: 430-438), Irritable bowel syndrome (ICD 9: 564.1), Depression (ICD-9: 296.2- 296.36 ICD-10: F32^*^, F33^*^, F34), Migraine (ICD-9: 346^*^), Dementia (ICD-9: 331.0-331.9; F00-F03, G30), Epilepsy (ICD-9: 345^*^) and MCI (ICD-10; F067).

### Statistical Analysis

Participant characteristics were computed to characterize the analytic source population and show differences between the groups with and without a T2D diagnosis for the study covariates. Next, schizophrenia rates by 10 000 person-years of follow-up were estimated.^26^

In the primary analysis, competing risk Cox regression models were fitted to estimate Hazard Ratios (HRs) for incident schizophrenia risk and to calculate their 2-sided 95% confidence intervals (CIs) with robust standard errors. This model, fitted as outlined^27^ and implemented^28^ elsewhere, accounted for the fact that during follow-up, mortality precludes a schizophrenia diagnosis, and followed-up to the event of schizophrenia. The models were implemented with age as the underlying timescale,^29^ based on their age at cohort entry following-up to the earliest age of schizophrenia diagnosis, leaving the HMO, all-cause mortality, or the end of follow-up, whichever came first. Two weighting procedures were applied to the primary Cox model. Inverse Probability Weights (IPWs) were applied to address time-dependent confounding, where covariates may be influenced by prior diabetes onset, and national post-stratification weights to improve generalizability (detailed in Supplementary Text S1, Table S2, Figure S3).

The groups with and without incident T2D were contrasted unadjusted and adjusted for the study covariates. For the unadjusted model, we estimated the cumulative incidence proportion^30^ and plotted the cumulative incidence curves to present the risk of incident schizophrenia for groups with and without T2D. The proportional hazards assumption was tested with the standard statistical test for the Cox model.^30^ The adjusted Cox model included all the covariates in the covariate section, fixed at cohort entry and time-varying, to control for measured confounding at each time point. In addition, we applied IPW to address time-dependent confounding, wherein prior diabetes exposure may influence subsequent covariates that also affect the outcome. By combining IPW with covariate adjustment in the outcome model, this approach accounts for the time-varying relationships between exposure, confounders, and outcome. This strategy strengthens confounding control and improves the stability and precision of the estimates. All models were fitted in R version 4.3.3 using the survival^30^ and IPW libraries.^31^

### Complementary Analysis

The robustness of the results from the primary analysis was challenged in 9 complementary analyses by altering the primary model. Sex differences in the association were examined in three ways. We included a sex × exposure interaction term in the Cox model to assess multiplicative interaction (model 1); conducted sex-stratified analyses to estimate sex-specific associations (model 2 and 3); and estimated the relative excess risk due to interaction (RERI) to assess additive interaction.

We considered the potential protective effects of antidiabetic medication (model 4). If T2D is associated with an increased schizophrenia risk, plausibly, antidiabetic medication, if effective in improving glycemic control or reducing systemic inflammation, may mitigate schizophrenia risk.^7^ In this analysis, restricted to individuals with diabetes, the primary model was refit to compare individuals exposed and unexposed to antidiabetic medication. Antidiabetic medication was analyzed as a time-varying covariate derived based on Anatomical Therapeutic Chemical (ATC) Classification System codes (ie, ATC code A10AB^*^). Antidiabetic medication purchases are only available to individuals by prescription from their HMO and are dispensed by pharmacies nationwide. All antidiabetic medications are heavily subsidized, and records are continuously updated upon purchase in the HMO electronic health records. Antidiabetic medications purchased based on a minimum time window of 45 days and a duration of at least 60 days were classified as Glucose (ATC code A10B^*^) or Insulin (ATC code: A10A^*^), otherwise unexposed.

To examine aspects of adult-onset schizophrenia, the outcome was changed to late−onset schizophrenia (onset age 50 up to 60 years; model 5) and then very−late−onset schizophrenia−like psychosis (age 60 and over; model 6).^14^ To consider the diagnostic reliability of schizophrenia, the primary model was refit removing individuals with a dementia diagnosis^21^ (model 7), and with cases restricted to over one schizophrenia diagnosis during follow-up (model 8).

To examine reverse causation, whereby early schizophrenia diagnoses following diabetes onset may reflect pre-existing or prodromal cases, we conducted sensitivity analysis like prior research (model 9).^15^ Specifically, we excluded schizophrenia cases that occurred within the first year after diabetes onset and then refitted the Cox model. The model incorporated IPW to and inverse probability of censoring weights. This approach allowed us to examine whether the association between diabetes and schizophrenia persisted after minimizing the influence of early events.

## Results

### Sample Characteristics

At cohort entry, the source population (n = 99 567) consisted of 52 517 (52.7%) females and 47 050 (47.3%) males and had a mean age of 59.70 (SD = 5.68). The mean follow-up time was 14.47 (SD = 2.28) years, and the mean age at the end of follow-up was 74.17 (SD = 5.65) years. The source population sample characteristics were computed on aggregate by T2D status (Table 1).

**Table 1:** Sample Characteristics

Covariate and classification	Overall N = 99 567	Diabetes absent n = 82 010 (82.37)	Diabetes present n = 17 557 (17.63)
**Age at cohort entry**	59.70 (5.68)	59.65 (5.69)	59.90 (5.62)
**Sex** Female	52 517 (52.7)	43 894 (53.5)	8623 (49.1)
Male	47 050 (47.3)	38 116 (46.5)	8934 (50.9)
**SES** Low	81 919 (82.3)	67 697 (82.5)	14 222 (81.0)
Medium	10 243 (10.3)	8534 (10.4)	1709 (9.7)
High	7405 (7.4)	5779 (7.0)	1626 (9.3)
**Smoking** No	79 222 (79.6)	67 257 (82.0)	11 965 (68.1)
Yes	20 345 (20.4)	14 753 (18.0)	5592 (31.9)
**Malnutrition** Absent	98 752 (99.2)	81 363 (99.2)	17 389 (99.0)
Present	815 (0.8)	647 (0.8)	168 (1.0)
**Obesity** Absent	81 369 (81.7)	70 529 (86.0)	10 840 (61.7)
Present	18 198 (18.3)	11 481 (14.0)	6717 (38.3)
**Asthma** Absent	87 954 (88.3)	73 526 (89.7)	14 428 (82.2)
Present	11 613 (11.7)	8484 (10.3)	3129 (17.8)
**COPD** Absent	94 827 (95.2)	78 715 (96.0)	16 112 (91.8)
Present	4740 (4.8)	3295 (4.0)	1445 (8.2)
**Hypertension** Absent	44 623 (44.8)	42 142 (51.4)	2481 (14.1)
Present	54 944 (55.2)	39 868 (48.6)	15 076 (85.9)
**IHD** Absent	82 545 (82.9)	70 665 (86.2)	11 880 (67.7)
Present	17 022 (17.1)	11 345 (13.8)	5677 (32.3)
**HF** Absent	94 128 (94.5)	78 665 (95.9)	15 463 (88.1)
Present	5439 (5.5)	3345 (4.1)	2094 (11.9)
**AF** Absent	91 566 (92.0)	76 358 (93.1)	15 208 (86.6)
Present	8001 (8.0)	5652 (6.9)	2349 (13.4)
**Cerebrovascular** Absent	86 078 (86.5)	72 402 (88.3)	13 676 (77.9)
Present	13 489 (13.5)	9608 (11.7)	3881 (22.1)
**IBS** Absent	94 076 (94.5)	77 724 (94.8)	16 352 (93.1)
Present	5491 (5.5)	4286 (5.2)	1205 (6.9)
**Depression** Absent	94 789 (95.2)	78 473 (95.7)	16 316 (92.9)
Present	4778 (4.8)	3537 (4.3)	1241 (7.1)
**Migraine** Absent	94 555 (95.0)	77 875 (95.0)	16 680 (95.0)
Present	5012 (5.0)	4135 (5.0)	877 (5.0)
**Dementia** Absent	93 255 (93.7)	77 386 (94.4)	15 869 (90.4)
Present	6312 (6.3)	4624 (5.6)	1688 (9.6)
**Epilepsy** Absent	98 182 (98.6)	80 903 (98.7)	17 279 (98.4)
Present	1385 (1.4)	1107 (1.3)	278 (1.6)
**MCI** Absent	98 447 (98.9)	81 184 (99.0)	17 263 (98.3)
Present	1120 (1.1)	826 (1.0)	294 (1.7)
**Insulin** Absent	98 599 (99.0)	82 010 (100.0)	16 589 (94.5)
Present	968 (1.0)	0 (0.0)	968 (5.5)
**Glucose** Absent	88 813 (89.2)	82 007 (100.0)	6806 (38.8)
Present	10 754 (10.8)	3 (0.0)	10 751 (61.2)
**Mortality** Absent	86 459 (86.8)	72 153 (88.0)	14 306 (81.5)
Present	13 108 (13.2)	9857 (12.0)	3251 (18.5)

From smoking to schizophrenia, the classification of the presence of a covariate is based on the presence at any time point during the study follow-up. Abbreviations. COPD, Chronic Obstructive Pulmonary Disease. AF, Atrial Fibrillation. HF, Heart Failure. IHD, Ischemic Heart Disease. TBI, Traumatic Brain Injury MCI, Mild Cognitive Impairment. IBS, Irritable Bowel Syndrome.

### Diabetes and Incident Adult Schizophrenia Risk

**Figure 2:**
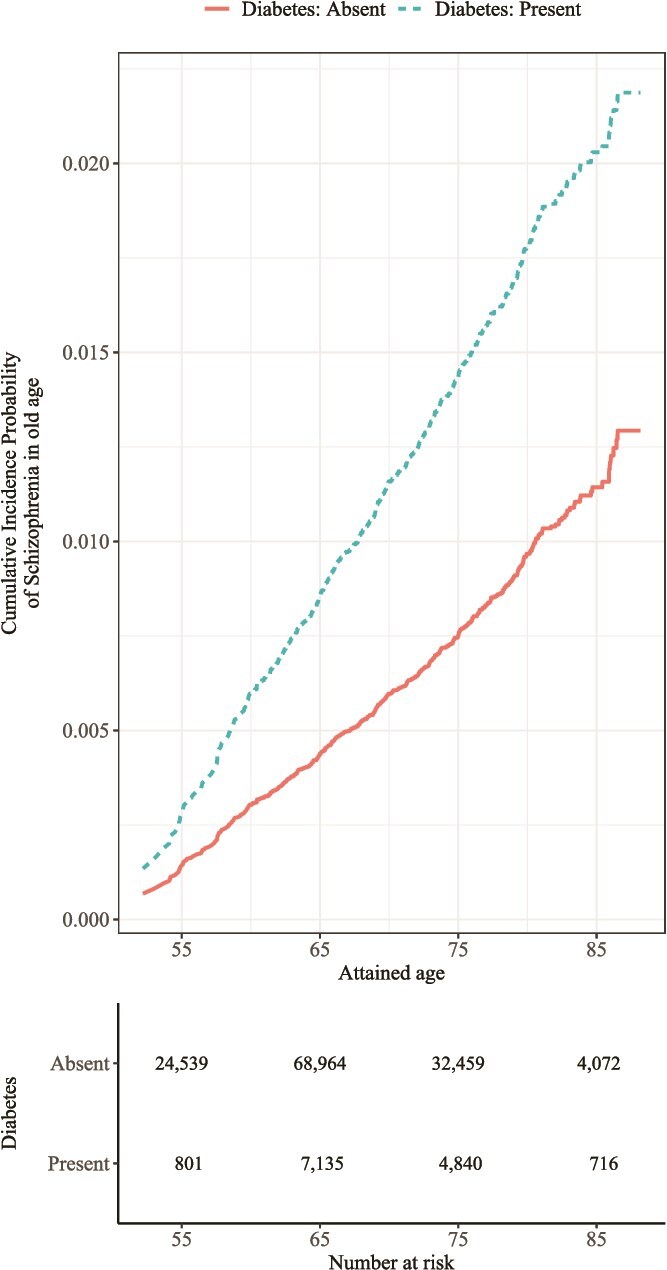
Cumulative Incidence Plot. The Cumulative Incidence Probability was Estimated Based on the Inverse Survival Plot Function from the Unadjusted Cox Regression Model.^30^ Below the Cumulative Incidence Curves are the Number At-Risk by Diabetes Status. As Shown in the Figure, Individuals Enter the Diabetes Cohort at Different Ages (Lowest Section). So, For Example, an Individual Entering the Study Cohort Diabetes-Free at Age 55, Diagnosed With Diabetes at Age 65, and Censored (for Death, Schizophrenia, the End of the Study, or Leaving the HMO) at age 70 Contributes to 10 years of Risk Time to the Adult Diabetes-Absent Group and 5 years of Risk Time to the Adult Diabetes Present Group.

During follow-up, the incident age of schizophrenia was (M = 68.36, SD = 7.63) significantly (t = –4.90, df = 561, *P*<.0001) older than T2D (M = 66.71, SD = 6.67). Although the crude cumulative proportions are similar (0.54%, Figure 1), they do not account for differences in follow-up time. To address this, incidence rates per person-years were calculated and estimated at 0.26 (95% CI, 0.21-0.32) per 10 000 person-years among individuals with incident T2D and 0.12 (95% CI, 0.11-0.14) among those without T2D (see Table S3 for the rates of the remaining covariates in the primary model). The cumulative incidence curves showed that the group with T2D present had higher incident adult schizophrenia-incident rates compared with T2D absent group (Figure 2).

**Figure 3:**
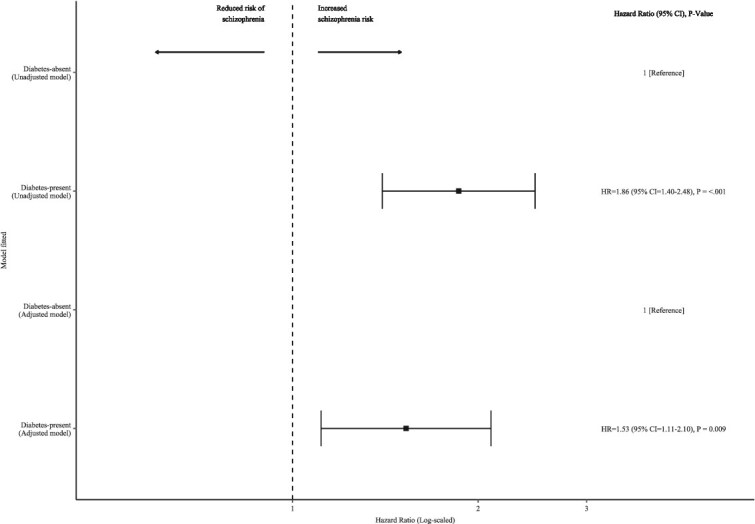
Primary Analysis: Type 2 Diabetes and the Risk of Schizophrenia. HR: Hazard Ratio from the Cox Regression Model, CI: Wald Two-Sided 95% Confidence Interval, *P*-value: *P*-value for Test of the Hypothesis HR = 1 vs the Hypothesis HR ≠ 1. Reference Group = Diabetes Absent.

There was no statistically significant deviation from the proportional hazards assumption for the association between T2D and incident adult schizophrenia risk (χ^2^ = 2.68, df = 1, *P*=.39). In the primary analysis, incident T2D was associated with an increased risk for incident schizophrenia (unadjusted: HR = 1.86 [95% CI = 1.40-2.48], *p* = <.001; adjusted HR = 1.53 [95% CI = 1.11-2.10], *P*=.009; Figure 3: adjusted model with all the covariates: Table S4) compared to the group without incident T2D.

### Complementary analyses

**Figure 4:**
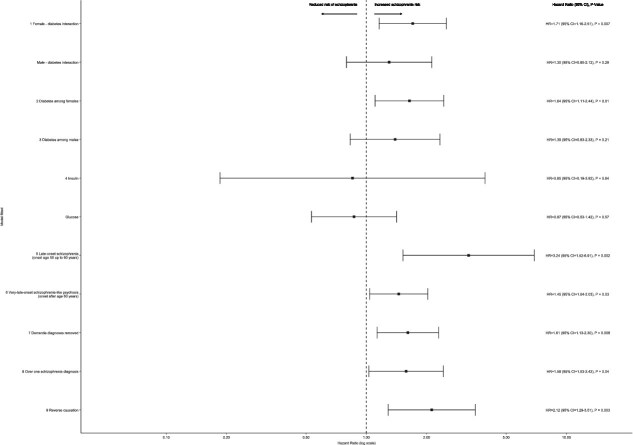
Complementary Analysis: Type 2 Diabetes and the Risk of Schizophrenia. HR: Hazard Ratio from the Cox Regression Model, CI: Wald Two-Sided 95% Confidence Interval, *P*-value: *P*-value for Test of the Hypothesis HR = 1 vs the Hypothesis HR ≠ 1. Reference Group = Diabetes Absent.

The robustness of the primary analysis was challenged in 9 complementary analyses by refitting the adjusted Cox model. Refitting the primary model to include a sex × diabetes interaction term, the association showed evidence of multiplicative interaction with a significant association between diabetes and schizophrenia among females (Figure 4, model 1). In models of sex-specific associations, among females, diabetes was statistically significantly associated with an increased risk of schizophrenia, but among males, the association was weaker and not statistically significant (Figure 4, models 2 and 3). The RERI was estimated at 0.41 (95% CI, –0.21 to 1.03), signaling a modest but null additive interaction.

In an analysis restricted to individuals with T2D, the group exposed to antidiabetic medication was not at greater risk of schizophrenia compared to the group unexposed (Figure 4, model 4). Altering the outcome to late−onset schizophrenia (model 5) and very−late−onset schizophrenia−like psychosis (model 6) did not attenuate the conclusion of the primary results. Refitting the primary model restricted to individuals without a dementia diagnosis (model 7) and to cases with over one schizophrenia diagnosis during follow-up (model 8), did not change the conclusion based on the primary analysis.

In the reverse causation model (Figure 4, Model 9), diabetes exposure was associated with a higher point estimate and smaller *P*-value for schizophrenia risk compared with the primary model (Figure 3), despite overlapping CI. This result suggests that the association is unlikely to be explained by reverse causation alone.

## Discussion

Based on a nationwide cohort study design, we examined the association between incident T2D and incident schizophrenia risk from midlife to old age. In this cohort, a diagnosis of T2D was associated with a 50% increased risk of incident very late-onset schizophrenia. Complementary analyses generally supported this association. Earlier studies have provided indirect evidence for the potential role of T2D in the etiology of schizophrenia. This includes evidence that the prevalence of diabetes is elevated in the year before the onset of schizophrenia^6^ and that in antipsychotic naïve first-episode psychosis, there is an increased prevalence of various abnormal glucose markers.^7^^,^^8^ Other evidence points to the shared familial risk for schizophrenia and T2D,^10^ increased T2D in siblings of individuals with schizophrenia,^9^ and genetic risk variants common to schizophrenia and T2D.^12^

Our study provides the strongest evidence to date in support of the hypothesis that T2D is associated with an increased risk of incident schizophrenia in middle and old age. One possible explanation for the association between T2D and schizophrenia incidence is that there are shared genetic factors^10^^,^^12^ influencing the risk for both T2D and schizophrenia. It may be that T2D is a specific risk factor related to schizophrenia onset in old age, thereby partly differentiating the etiologies of schizophrenia by early adult life and from old age.^14^ Another possible explanation is through common stress-related processes^32^ given that stress is a potential risk factor for schizophrenia and T2D in adults.^32^^,^^33^ Furthermore, T2D is associated with decrements in cognitive function and changes in brain structures known to be associated with schizophrenia.^34^ Poor cognitive functioning is a core symptom of schizophrenia, and cognitive decline predates the onset of schizophrenia.^35^ Plausibly, in some patients, the onset of diabetes leads to a cascade of downstream changes in the brain that could trigger psychosis. Possibly, individuals with T2D who later develop schizophrenia already exhibit early, subclinical symptoms of the disorder. Such prodromal features may contribute to poor self-care, inadequate nutrition, and metabolic instability, potentially accelerating schizophrenia onset.

Diabetes was associated with a higher risk of late onset schizophrenia in women. This finding may be of clinical significance since the incidence of old-age schizophrenia is higher among females than males.^36^ Multiple tentative mechanisms, such as estrogen loss, may be postulated for this association among females.^37^ Nonetheless, while a signal toward increased risk was observed among males, it was not statistically significant, possibly due to the small number of males with diabetes and schizophrenia. Future research with larger samples of males is warranted for firmer conclusions regarding sex differences in the association. Among individuals with T2D additional factors, such as loneliness and social isolation, may also contribute to sex differences in the risk of schizophrenia.

### Limitations and Implications

To our knowledge, this is the first study to examine the association between incident T2D and schizophrenia incidence in midlife to old age. We used a nationwide cohort study design in a setting where selection would be illegal, harnessed on a large sample size with up to 15 years of follow-up, and aimed to account for 20 sources of confounding and channeling bias.

However, our study has several limitations. We lacked information about possible earlier life exposure to antipsychotic medication, which may increase the risk for T2D. However, this bias seems unlikely since we excluded individuals for three years before cohort entry with schizophrenia. Nonetheless, a longer exclusion period may be have been desirable. Furthermore, schizophrenia could be under-ascertained, yet the rates in our population data are similar to some other studies.^38^ However, compared to some other studies, our rate may underestimate schizophrenia,^39^^,^^40^ suggesting that our risk estimate is an underestimate. In addition, we lacked information on the onset of schizophrenia by early adulthood. Equally, caution is warranted before extrapolating our results to the onset of schizophrenia by early adulthood.^14^ Such information may be useful in distinguishing the role of T2D in early and late-onset etiologies.

We were unable to ascertain specific symptoms of schizophrenia with a clinical measure such as the PANSS, hence, positive vs negative symptoms could not be assessed. We lacked information about potential sources of confounding, including the genetic risk for schizophrenia and T2D. We also lacked potentially important demographic information, such as ethnic or migrant status. Accordingly, residual confounding may have occurred, although it is unlikely it would attenuate our findings to null. Equally, we did not have information on HBA1C levels and so could not test hypotheses regarding the severity of T2D and schizophrenia. It is possible that individuals with T2D are more likely to be forwarded for or seek care for other disorders (ie, Berkson’s bias may have occurred).^41^ To address this bias, we used inverse probability weights to adjust for selection bias that may distort the association between T2D and schizophrenia.

Future research is warranted to examine the association between diabetes and the course of schizophrenia. Particularly, to examine individuals whose schizophrenia onset was associated with diabetes compared with a suitable comparison group, and the course of psychiatric and diabetic medication and hospitalization. Similarly, we could not examine diagnoses of T2D and coma based on these community-based data, due to the small sample size with this diagnosis. This and the exacerbation of T2D may be an avenue for future research.

We examined the potential role of modification by antidiabetic medications in sensitivity analysis. We observed a non-significant difference between (glucose and insulin) antidiabetic medication and reduced schizophrenia risk. Perhaps this reflects statistical power or a lack of the newer antidiabetic medications (eg, SGLT2 inhibitors) in our data that offer greater risk modifying potential than glucose and insulin. Further research remains necessary to examine the association between antidiabetic medication and schizophrenia incidence. It is important to note that our study data and analysis do not permit a causal interpretation of the association between T2D and schizophrenia.

## Conclusions

In this nationwide cohort study comprised of 99 567 adults with up to 15 years of follow-up, findings showed that the onset of T2D in adulthood was associated with a 50% increased risk of new-onset schizophrenia in mid- and old-age. This finding was generally supported by complementary analyses considering diagnostic aspects of schizophrenia and diabetes; and reverse causation was minimal. Policymakers, healthcare providers, clinicians, patients, and caregivers may wish to consider monitoring for psychosis from midlife in individuals with T2D.

## Supplementary Material

R1_Supplementary_Information_sbaf159
