# Supplementary material for: The Risk of Very Late-Onset Schizophrenia Following Diabetes Type 2 Onset: A Nationwide Population-Based Study of Midlife and Old-Age
Source: Schizophr Bull. 2025 Sep 13;52(4):sbaf159. doi: 10.1093/schbul/sbaf159 (PMC13391628; doi:10.1093/schbul/sbaf159)
Supplement: R1_Supplementary_Information_sbaf159 [file r1_supplementary_information_sbaf159.docx]

Suppmement to Levine, Kodesh and Reichenberg. **The risk of schizophrenia following diabetes type 2 onset: A nationwide population-based study**

[Text S1 Weighting Schemes in the Primary Analysis 2](#_Toc202708110)

[Table S1 Diabetes Diagnoses 3](#_Toc202708111)

[Table S2 Age-by-sex distribution in the study sample and national population, and corresponding post-stratification weights 4](#_Toc202708112)

[Table S3 Rates per 10,000 person-years 5](#_Toc202708113)

[Table S4 Primary model including all covariates 6](#_Toc202708114)

[Figure S1 Reverse causation illustrated 7](#_Toc202708115)

[Figure S2 Follow-up time illustration 8](#_Toc202708116)

[Figure S3 Distribution of stabilized inverse probability weights 9](#_Toc202708117)

[References S1 10](#_Toc202708118)

# Text S1 Weighting Schemes in the Primary Analysis

Two types of weighting procedures were used in the primary Cox model. First, inverse probability weights (IPW) were applied to address time-dependent confounding bias, where covariates may be influenced by prior diabetes onset. The IPW model included all the study covariates, both at cohort entry and time-varying, to estimate the probability of exposure at each time point. Stabilized weights, derived from these covariates, were used to improve precision and reduce variance. These weights were well-distributed (range, 0.18–6.18; mean, 1.00; SD, 0.21), indicating good stability and minimal risk of bias from extreme values (Supplementary Figure S3). Covariates, including time-varying variables, were included directly in the Cox models to account for measured confounding. Combined with stabilized IPW, this approach strengthened confounding control and minimized residual bias. Second, national post-stratification weights were applied to address potential selection bias due to the limitations in the coverage of Meuhedet (14% of the Israeli population; Supplementary Table S2) and to improve generalizability. These were calculated as the ratio of the national population proportion to the corresponding proportion in the analytic sample. As shown in Supplementary Table S2, several strata were underrepresented and overrepresented. Post-stratification weights ranged from 0.71 to 2.00 (median = 0.86, mean = 1.00), indicating moderate adjustment without the introduction of extreme weights. These two weighting strategies were used to address internal and external validity, respectively: IPW and national post-stratification weights. Their product was applied as a combined weight in the primary Cox regression models.

# Table S1 Diabetes Diagnoses

| 250.00 Diabetes mellitus without mention of complication, type II or unspecified type, not stated as uncontrolled |
| --- |
| 250.02 Diabetes mellitus without mention of complication, type II or unspecified type, uncontrolled |
| 250.10 Diabetes with ketoacidosis, type II or unspecified type, not stated as uncontrolled |
| 250.12 Diabetes with ketoacidosis, type II or unspecified type, uncontrolled |
| 250.20 Diabetes with hyperosmolarity, type II or unspecified type, not stated as uncontrolled |
| 250.22 Diabetes with hyperosmolarity, type II or unspecified type, uncontrolled |
| 250.30 Diabetes with other coma, type II or unspecified type, not stated as uncontrolled |
| 250.32 Diabetes with other coma, type II or unspecified type, uncontrolled |
| 250.40 Diabetes with renal manifestations, type II or unspecified type, not stated as uncontrolled |
| 250.42 Diabetes with renal manifestations, type II or unspecified type, uncontrolled |
| 250.50 Diabetes with ophthalmic manifestations, type II or unspecified type, not stated as uncontrolled |
| 250.52 Diabetes with ophthalmic manifestations, type II or unspecified type, uncontrolled |
| 250.60 Diabetes with neurological manifestations, type II or unspecified type, not stated as uncontrolled |
| 250.62 Diabetes with neurological manifestations, type II or unspecified type, uncontrolled |
| 250.70 Diabetes with peripheral circulatory disorders, type II or unspecified type, not stated as uncontrolled |
| 250.72 Diabetes with peripheral circulatory disorders, type II or unspecified type, uncontrolled |
| 250.80 Diabetes with other specified manifestations, type II or unspecified type, not stated as uncontrolled |
| 250.82 Diabetes with other specified manifestations, type II or unspecified type, uncontrolled |
| 250.90 Diabetes with unspecified complication, type II or unspecified type, not stated as uncontrolled |
| 250.92 Diabetes with unspecified complication, type II or unspecified type, uncontrolled |

Note. Based on ICD-9 codes.(1)

# Table S2 Age-by-sex distribution in the study sample and national population, and corresponding post-stratification weights

| Age Group | Sex | Population N(%) | Study N(%) | Population % | Study % | Weight |
| --- | --- | --- | --- | --- | --- | --- |
| 51–55 | Female | 136000 (14.6%) | 17710 (17.8%) | 14.6 | 17.8 | 0.82 |
| 51–55 | Male | 126700 (13.6%) | 15816 (15.9%) | 13.6 | 15.9 | 0.86 |
| 56–60 | Female | 107300 (11.6%) | 16374 (16.4%) | 11.6 | 16.4 | 0.71 |
| 56–60 | Male | 99200 (10.7%) | 14863 (14.9%) | 10.7 | 14.9 | 0.72 |
| 61–65 | Female | 113400 (12.2%) | 8954 (9.0%) | 12.2 | 9.0 | 1.36 |
| 61–65 | Male | 97500 (10.5%) | 8075 (8.1%) | 10.5 | 8.1 | 1.30 |
| 66–70 | Female | 99100 (10.7%) | 7430 (7.5%) | 10.7 | 7.5 | 1.43 |
| 66–70 | Male | 81100 (8.7%) | 6571 (6.6%) | 8.7 | 6.6 | 1.32 |
| 71+ | Female | 38800 (4.2%) | 2049 (2.1%) | 4.2 | 2.1 | 2.00 |
| 71+ | Male | 29800 (3.2%) | 1725 (1.7%) | 3.2 | 1.7 | 1.88 |

Note. National and study percentages reflect the proportion of individuals in each age-by-sex stratum relative to their respective total populations. Post-stratification weights were calculated as the ratio of the national population percentage to the study sample percentage for each stratum. Weights greater than 1 indicate under-representation in the study sample and increase the influence of those strata; weights less than 1 indicate over-representation and reduce the influence accordingly. Calculated weights ranged from 0.54 to 2.15 (median = 0.86, mean = 1.00), indicating moderate adjustment to correct for discrepancies without introducing extreme weights.

# Table S3 Rates per 10,000 person-years

| **Covariate** | **Classification** | **Rate (95% CI)** |
| --- | --- | --- |
| Sex | Female | 0.17 (0.15,0.18) |
|  | Male | 0.13 (0.11,0.14) |
| Socioeconomic status | High | 0.12 (0.09,0.17) |
|  | Low | 0.14 (0.13,0.16) |
|  | Medium | 0.19 (0.15,0.24) |
| Smoking status | Absent | 0.14 (0.13,0.16) |
|  | Present | 0.21 (0.16,0.27) |
| Malnutrition | Absent | 0.15 (0.13,0.16) |
|  | Present | 0.55 (0.21,1.15) |
| Obesity | Absent | 0.14 (0.13,0.15) |
|  | Present | 0.21 (0.17,0.26) |
| Asthma | Absent | 0.14 (0.13,0.15) |
|  | Present | 0.23 (0.18,0.30) |
| COPD | Absent | 0.15 (0.13,0.16) |
|  | Present | 0.26 (0.17,0.39) |
| Hypertension | Absent | 0.12 (0.11,0.14) |
|  | Present | 0.18 (0.16,0.20) |
| IHD | Absent | 0.15 (0.13,0.16) |
|  | Present | 0.17 (0.13,0.21) |
| HF | Absent | 0.14 (0.13,0.16) |
|  | Present | 0.33 (0.22,0.46) |
| AF | Absent | 0.14 (0.13,0.16) |
|  | Present | 0.26 (0.19,0.35) |
| Cerebrovascular diseases | Absent | 0.14 (0.13,0.15) |
|  | Present | 0.29 (0.23,0.36) |
| IBS | Absent | 0.14 (0.13,0.16) |
|  | Present | 0.23 (0.16,0.33) |
| Depression | Absent | 0.13 (0.12,0.14) |
|  | Present | 0.87 (0.69,1.09) |
| Migraine | Absent | 0.15 (0.14,0.16) |
|  | Present | 0.14 (0.09,0.23) |
| Dementia | Absent | 0.11 (0.10,0.13) |
|  | Present | 1.48 (1.24,1.74) |
| Epilepsy | Absent | 0.15 (0.13,0.16) |
|  | Present | 0.45 (0.23,0.77) |
| MCI | Absent | 0.14 (0.13,0.16) |
|  | Present | 1.10 (0.62,1.79) |
| Diabetes | Absent | 0.14 (0.12,0.15) |
|  | Present | 0.27 (0.22,0.33) |

Note. Abbreviations. COPD, Chronic Obstructive Pulmonary Disease. IHD, Ischemic Heart Disease. HF, Heart Failure. AF, Atrial Fibrillation. IBS, Irritable bowel syndrome. MCI, Mild Cognitive Impairment. CI, 95% CI Confidence Intervals. Rates weigted by the national population.

# Table S4 Primary model including all covariates

| **term** | **HR (95% CI), P-value** |
| --- | --- |
| Age at cohort entry (linear) | 0.73 (0.48- 1.09), 0.12 |
| Age at cohort entry (quadratic) | 1.00 (1.00- 1.01), 0.06 |
| Sex (male) | 0.86 (0.71- 1.04), 0.13 |
| Ses (medium) | 1.47 (0.93- 2.31), 0.10 |
| Ses (low) | 1.00 (0.68- 1.46), 0.98 |
| Smoking | 1.33 (0.92- 1.92), 0.13 |
| Malnutrition | 1.63 (0.59- 4.46), 0.34 |
| Obesity | 1.14 (0.87- 1.50), 0.33 |
| Asthma | 1.22 (0.89- 1.66), 0.21 |
| Copd | 1.18 (0.70- 1.99), 0.53 |
| Hypertension | 0.96 (0.76- 1.20), 0.71 |
| Ihd | 0.76 (0.55- 1.06), 0.10 |
| Hf | 1.16 (0.69- 1.93), 0.57 |
| Af | 1.13 (0.71- 1.81), 0.61 |
| Cerebrovascular diseases | 0.99 (0.70- 1.38), 0.94 |
| Ibs | 0.90 (0.58- 1.41), 0.65 |
| Depression | 3.61 (2.51- 5.19), <.001 |
| Migraine | 0.95 (0.54- 1.70), 0.87 |
| Dementia | 10.78 (7.64-15.21), <.001 |
| Epilepsy | 1.15 (0.60- 2.19), 0.67 |
| Mci | 1.11 (0.59- 2.09), 0.74 |
| Diabetes | 1.53 (1.11- 2.10), 0.009 |

Note. Abbreviations. COPD, Chronic Obstructive Pulmonary Disease. IHD, Ischemic Heart Disease. HF, Heart Failure. AF, Atrial Fibrillation. IBS, Irritable bowel syndrome. MCI, Mild Cognitive Impairment.

CI, 95% CI Confidence Intervals. The reference group for sex is female sex, for SES, low SES, and from Smoking status to diabetes, the covariate is time-dependent, and the reference group is classified as disorder absent.

# Figure S1 Reverse causation illustrated


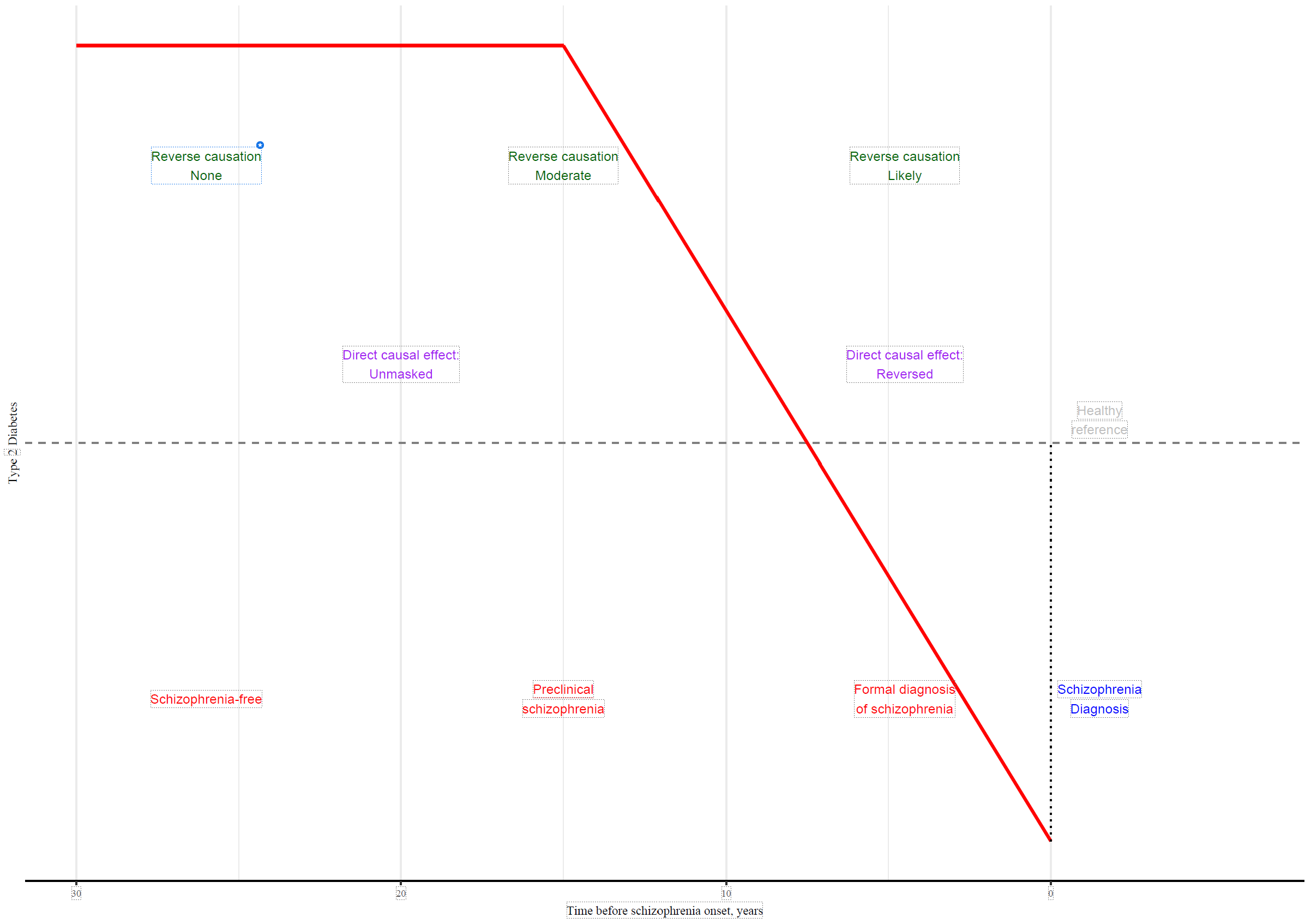


Note. Supplementary Figure 1 was adapted from Kivimäki, Luukkonen, Batty, Ferrie, Pentti, Nyberg, et al.(2) To examine reverse causation, the analysis was stratified by sequential duration of follow-up. Based on three successive five-year intervals, the associations between diabetes and schizophrenia risk were

scrutinized. Type 2 Diabetes (T2D) was assumed to have less of an effect on schizophrenia risk in the preclinical schizophrenia stage when the diagnosis was long before schizophrenia onset and considerably affected risk when the T2D was nearer the diagnosis. Therefore, a stronger association in the first intervals of follow-up time may suggest that reverse causation occurs.

# Figure S2 Follow-up time illustration


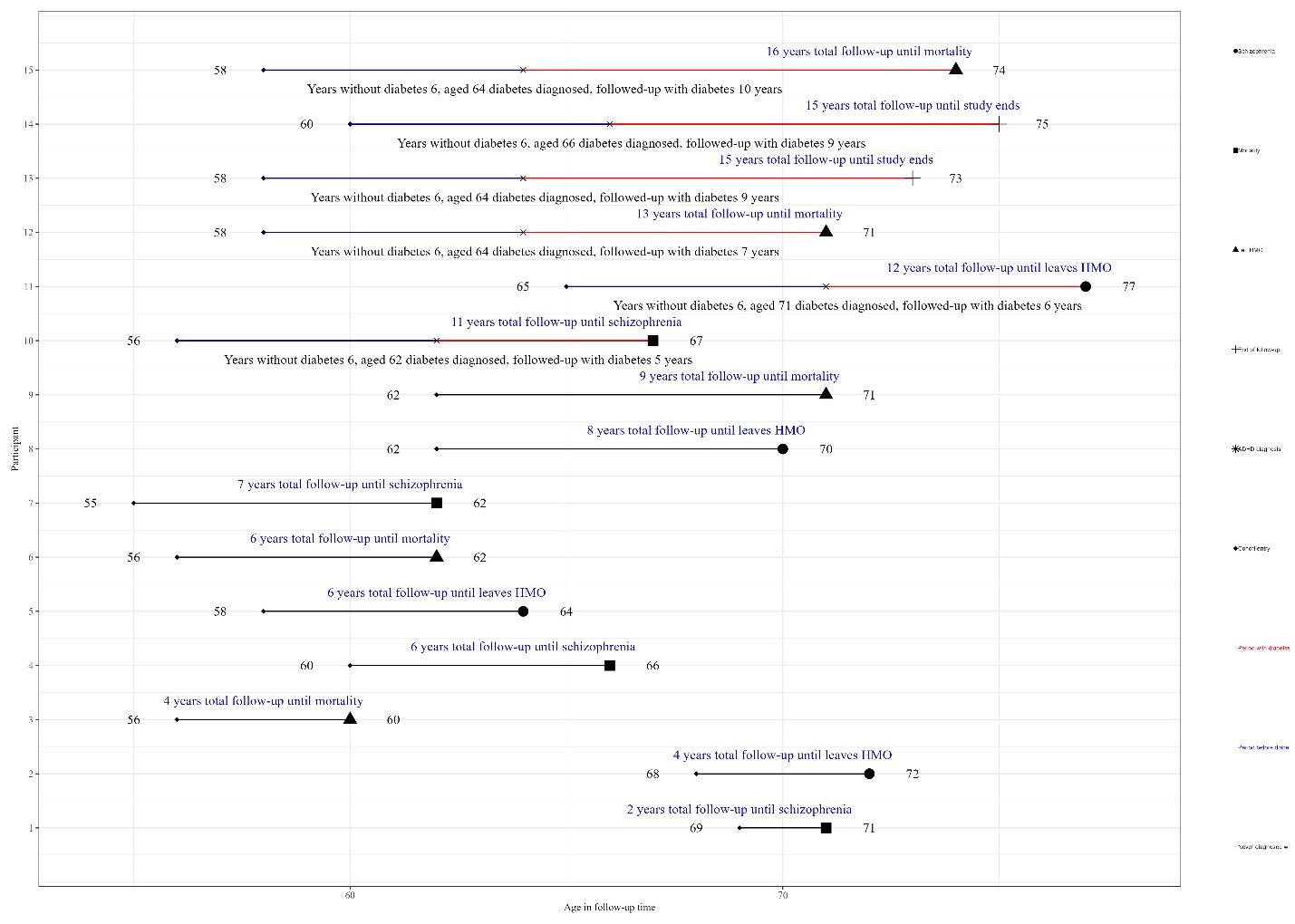
Note. eFigure 2 illustrates the cohort entry and end ages of follow-up for 15 fictitious study participants. From the left, we can observe the ages when participants entered the cohort (age and diamond) and to the far right when censored and follow-up ended. Above the line of each participant in blue is the number of years at risk with the reason follow-up ended (Circle: leaving the HMO; Triangle: all-cause mortality; Square: Schizophrenia; Cross: end of study follow-up). Participants 10 to 15 illustrate the time-varying nature of adult diabetes. For these participants, a blue line indicates their periods without a diabetes diagnosis, the age at diabetes diagnosis (indicated by a star), and a red line represents diabetes present during follow-up. For instance, participant 14 began the study at age 60, was followed up for six years without diabetes, was diagnosed with diabetes at age 66, and was followed-up with diabetes for a further 15 years and completed the total study follow-up.

# Figure S3 Distribution of stabilized inverse probability weights


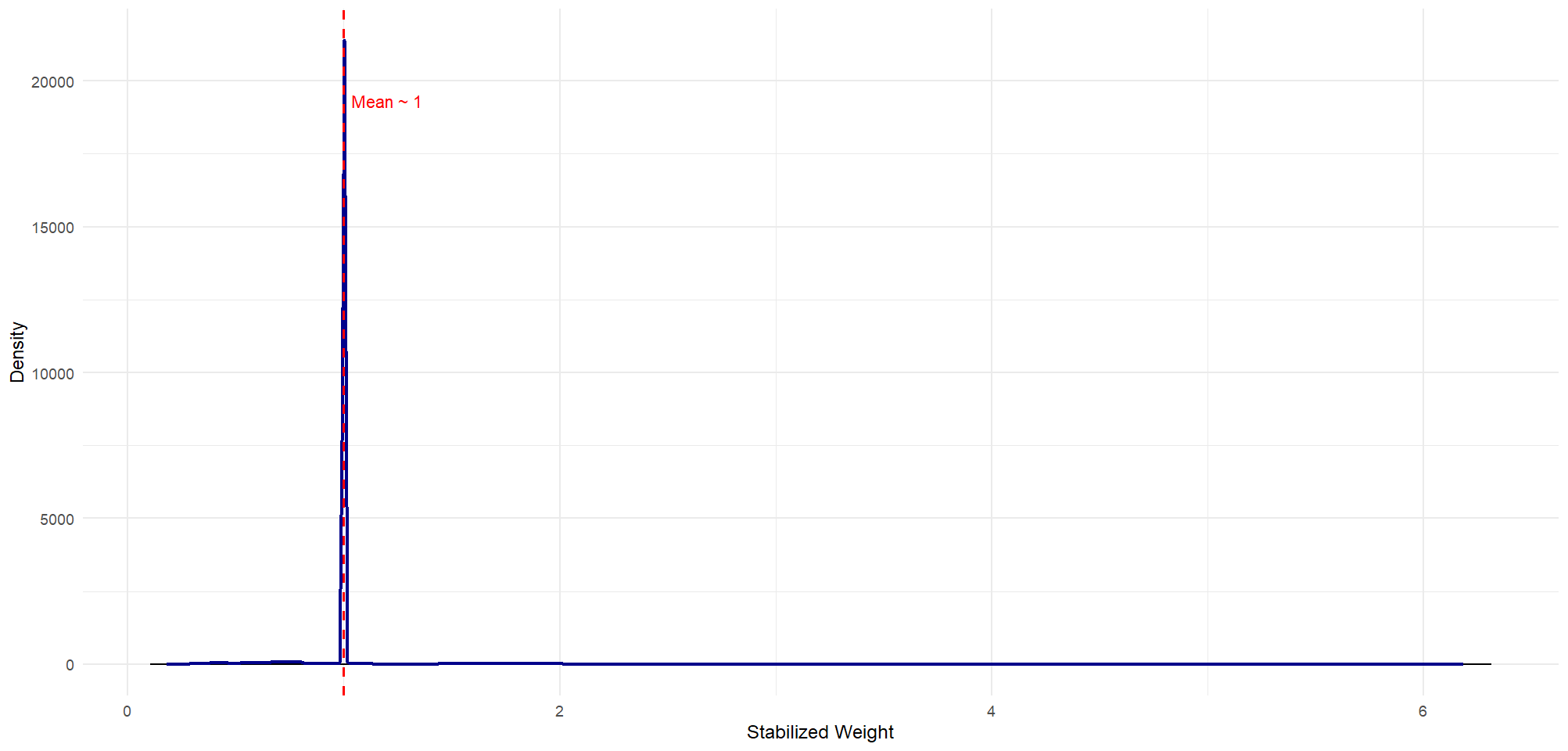


Note. Weights ranged from 0.18 to 6.18 (mean, 1.00; SD, 0.21), with most values tightly clustered near 1.0. The distribution indicates modest correction for time-dependent confounding without introducing extreme or unstable values. This histogram with an overlaid density curve illustrates the weight distribution.

# References S1

1. World Health Organization: International Classification of Diseases,Ninth Revision, Clinical Modification (ICD-9-CM). Medicode, Salt Lake City (UT) 2000

2. Kivimäki M, Luukkonen R, Batty GD, et al.: Body mass index and risk of dementia: Analysis of individual-level data from 1.3 million individuals. Alzheimers Dement 14:601-9, 2018
